# Endocrinopathies and Male Infertility

**DOI:** 10.3390/life12010010

**Published:** 2021-12-22

**Authors:** Pallav Sengupta, Sulagna Dutta, Ivan Rolland Karkada, Suresh V. Chinni

**Affiliations:** 1 Physiology Unit, Faculty of Medicine, Bioscience and Nursing, MAHSA University, Jenjarom 42610, Malaysia; pallav@mahsa.edu.my (P.S.); ivanroland@mahsa.edu.my (I.R.K.); 2 School of Medical Sciences, Bharath Institute of Higher Education and Research (BIHER), Chennai 600126, India; duttasulagna@mahsa.edu.my; 3 Department of Oral Biology and Biomedical Sciences, Faculty of Dentistry, MAHSA University, Jenjarom 42610, Malaysia; 4 Department of Biotechnology, Faculty of Applied Sciences, AIMST University, Bedong 08100, Malaysia

**Keywords:** endocrinopathies, male infertility, hypothyroidism, hyperprolactinemia

## Abstract

Male infertility is approaching a concerning prevalence worldwide, and inflicts various impacts on the affected couple. The hormonal assessment is a vital component of male fertility evaluation as endocrine disorders are markedly reversible causatives of male infertility. Precise hormonal regulations are prerequisites to maintain normal male fertility parameters. The core male reproductive event, spermatogenesis, entails adequate testosterone concentration, which is produced via steroidogenesis in the Leydig cells. Physiological levels of both the gonadotropins are needed to achieve normal testicular functions. The hypothalamus-derived gonadotropin-releasing hormone (GnRH) is considered the supreme inducer of the gonadotropins and thereby the subsequent endocrine reproductive events. This hypothalamic–pituitary–gonadal (HPG) axis may be modulated by the thyroidal or adrenal axis and numerous other reproductive and nonreproductive hormones. Disruption of this fine hormonal balance and their crosstalk leads to a spectrum of endocrinopathies, inducing subfertility or infertility in men. This review article will discuss the most essential endocrinopathies associated with male factor infertility to aid precise understanding of the endocrine disruptions-mediated male infertility to encourage further research to reveal the detailed etiology of male infertility and perhaps to develop more customized therapies for endocrinopathy-induced male infertility.

## 1. Introduction

Male infertility has stirred global concerns over the trend of its increasing prevalence and ambiguity of its etiopathogenesis [1,2,3,4,5,6]. Given that almost half of the global infertility cases involve male factors, it is essential to understand the core mechanisms of male infertility causation [7]. The endocrine system is the prime regulator of the reproductive functions [8,9,10]. Male reproductive functions are dependent on a complex crosstalk of hormones [10]. Gonadotropin-releasing hormone (GnRH) is synthesized by the hypothalamus and promotes the anterior pituitary to secrete the gonadotropins, luteinizing hormone (LH) and follicle-stimulating hormone (FSH). FSH acts on the Sertoli cells, which accelerate the spermatogonial maturation. In Leydig cells, LH acts to cause the synthesis and release of testosterone [9,10]. The testicular testosterone level must be much greater than that in the serum, to support normal spermatogenesis. This intratesticular testosterone indirectly increases germ cell maturation as a result of its actions on Sertoli cells [11,12]. Despite the fact that endocrinopathies are only occasionally related with infertility in males (about 1% to 2%), the treatment of these disorders provides patients with a tailored approach to fertility preservation and restoration [12]. Endocrinopathies can be divided into two categories: those characterized by a lack of hormones and those characterized by an excess of hormones [13]. In each of these categories, specific hormonal abnormalities fall into one of the subcategories with specific hormonal abnormalities (Table 1; [12,14,15,16,17,18,19,20,21,22,23,24,25,26,27,28]). In this review, we elucidate the disorders in which hormone imbalance can have a deleterious impact on male fertility. We go over the pathogenesis and clinical presentations of each endocrine disorder extensively. We also enumerate the standard diagnosis procedures as well as the ideal management approach, as well as prospective future areas for study in this field.

## 2. Hypogonadotropic Hypogonadism

A state of reduced testosterone production caused by low levels of the gonadotropins and estradiol is known as hypogonadotropic hypogonadism (HH). Many different factors can contribute to HH, which can be classified into two groups: congenital and acquired GnRH deficiency [14].

The Kallmann syndrome is a genetic etiology of HH. This genetic disorder occurs in an X-linked recessive manner [29]. It can be caused by diverse mutations, the most common is KAL1 gene mutation. There are several characteristics of hypogonadism, including facial deformities, anosmia, neurologic abnormalities and renal agenesis [15,30,31]. Hypogonadism and its clinical implications (including delayed puberty and infertility) are caused by a malfunction of GnRH-secreting neurons to migrate [12]. As a result of this failure of migration, GnRH secretion is absent, which again results in the cessation of gonadotropin secretion. In pituitary functional disruption caused by tumors, surgery, stroke, or infiltrative disease, HH can be acquired rather than genetically inherited [14]. Whatever the underlying cause of HH, the fundamental problem is low gonadotropin levels, which can be corrected with pharmacological replacement [32]. Gonadotropin therapy (GT) is used to treat HH, which is characterized by the replacement of insufficient hormones. It is administered with the help of hormones such as human menopausal gonadotropin (hMG), recombinant FSH (rFSH), and human chorionic gonadotropin (hCG). The utilization of hCG originates due to its qualities as an LH analogue, which act on the Leydig cells, increasing the synthesis of androgens throughout the reproductive process [32]. hMG is a hormone found in postmenopausal women urine samples, which contain both the gonadotropins. It is used to treat menopausal symptoms [33]. For men with HH, GT is often commenced by administration of hCG alone for 3 to 6 months before inclusion of other hormones. The doses of hormones range from 1000 to 1500 USP units administered intravenously or subcutaneously thrice weekly. The effectiveness of treatment can be determined by testing serum testosterone levels, for maintaining its normal levels over time. When it comes to spermatogenesis, appropriate intratesticular testosterone concentrations are the most important goal to achieve. However, it is not routinely measured in GT. Testicular testosterone concentrations, on the other hand, indicate a linear relationship with the amount of hCG injected [15]. Typically, after 3 to 6 months of hCG monotherapy, the patient’s testosterone levels stabilize and the patient becomes ready to begin replacement medication for FSH levels. One technique of FSH replacement comprises the administration of hMG at doses ranging from 75 to 150 IU IM/SC thrice weekly at different body sites. As an alternative, rFSH be administered at 150 IU IM/SC thrice weekly in combination with other hormones [5]. There are few studies on the relative efficacy of hMG as compared to rFSH in women undergoing in vitro fertilization (IVF), but there has been very little research done in male patients. It has been demonstrated that this method of replacing gonadotropins produces good results, with spermatogenesis occurring in more than 90% of treated males [12]. In most cases, the time it takes for spermatogenesis to occur is fairly variable, with the average response occurring in 6 to 9 months on average. Individuals may need to be treated for up to 12 years before seeing any improvement, and some may never see any improvement with this treatment [34]. A study conducted on 38 Australian men with HH showed that the median period from first sperm in the ejaculate to conception was 7.1 months [31]. Despite the fact that spermatogenesis takes place in a great proportion of infertile patients, sperm concentrations reached via GT can still fall below target levels (often less than 20 × 10^6^ per mL). In spite of this fact, the GT leads to extremely favorable fertility outcomes. In another study, 24 men with HH treated with gonadotropin had the mean sperm concentration of 16.7 × 10^6^ per mL and still they achieved pregnancy [35]. Men with testicles larger than prepubertal sizes (>4 mL) were found to have higher rates of sperm production, according to retrospective research of Japanese men [36]. A total of 87 infertile males with HH were researched in Saudi Arabia. They were administered with intramuscular gonadotropins for 26 months, improving fertility in the study group. As a whole, 35 of the 87 patients (40%) were successful in conceiving their child [37]. A substantial number of studies focus on establishing determinants of response to GT, which is an essential topic of investigation. According to the results of the previously described long-duration research on Japanese men, there is a relationship between the pretreatment and post-GT testicular size. According to a study, males with testicular sizes greater than 4 mL bore 71% likelihood to respond of treatment, while men with testis sizes less than 4 mL showed about 36% possibility to respond to the treatment. Furthermore, the above-mentioned study discovered that just the size of the testicles prior to treatment was a conception determinant. Specifically, men who responded to the treatment showed a testis volume of 9.0 ± 3.6 mL prior to treatment, whereas for the nonresponders, the mean testicular volume was 5.7 ± 2.0 mL prior to treatment [37]. It is important to note that the variations in conception rates between males who suffered from HH caused by congenital or acquired etiologies are not statistically significant. A greater size of testis was described to be an individual marker of response time to GT, and attaining a total testicular volume of over 20 mL following treatment doubled the chance of attaining both the normal semen quality and conception rate [31,35]. The lower sperm concentrations than the typical targets of infertility therapy, shown in these trials, may be able to attain pregnancy when the treatment follows appropriate assisted reproductive therapies (ARTs). Aside from that, such clinical management may improve the effectiveness of sperm extraction. An additional therapy option for men suffering from HH is the practice of antiestrogen medications. These compounds attach with hypothalamic estrogen receptors (ER) in a competitive manner. When estradiol is present, it inhibits gonadotropin release at this endocrine site through the process of ‘negative feedback’.

Antiestrogen drugs work by binding to these receptors in the hypothalamus, preventing hypothalamic negative feedback of estradiol and resulting in an upsurge in GnRH release from the hypothalamus. Higher GnRH secretion results in elevated adenohypophyseal gonadotropin secretion, which in turn drives an upsurge in testicular testosterone synthesis. In this family of drugs, clomiphene citrate is the most often used agent, but other related drugs include raloxifene, tamoxifen, and toremifene. These medications have been explored in the context of empirical treatment for idiopathic male infertility in the past, with variable outcomes [31,34]. The focused use of clomiphene in individuals with established HH, on the other hand, has proven to be beneficial in a few specific situations. Four men suffering from HH were administered with 50 mg clomiphene citrate thrice weekly in a study conducted in the United States, and three of the patients had better testosterone levels and semen parameters. Two of these three males went on to have documented pregnancies as a result of this [38]. Case studies have also reported alike improvements at the biochemical level, despite the fact that reproduction was not the primary target of these therapies [18,39]. One of the retrospective studies involving 31 men examined the efficacy of clomiphene citrate in comparison to androgen therapy. The highest serum testosterone levels were found in the group that received testosterone injections, while the lowest levels were seen in the groups who received topical testosterone plus clomiphene treatment. However, it was confirmed by the Androgen Deficiency in Aging Males (ADAM) questionnaire that there was no difference in overall levels of satisfaction across the different groups [40,41]. The usage of clomiphene for the management of male infertility has been linked with diverse adversities, including visual problems, gastrointestinal distress, weight gain, hypertension, and sleeplessness [34]. Despite the fact that administering testosterone therapy to promote spermatogenesis is not suggested, a substantial number of healthcare providers continue to employ this strategy to this purpose. It was discovered in a study that almost 25% of urologists examined have recommended testosterone to increase spermatogenesis [41]. Exogenous testosterone, on the other hand, has been shown to have negative effects on spermatogenesis. An increase in exogenous testosterone triggers hypothalamic negative feedback, resulting in reduced levels of GnRH, LH, and FSH, and also testicular testosterone. It has been reported that suppressing testicular testosterone < 20 ng/mL can significantly affect spermatogenesis [42]. Exogenous GnRH medication is an additional medical treatment option for HH that might be explored. Synthetic GnRH analogues can be injected to induce the secretion of gonadotropins. However, because of the shorter half-life of these agents, as well as the requirement for pulsatile secretion, a method of frequent administration must be used to administer them. These approaches are inconvenient, and evidences show no significant improvement via treatment of HH [11].

## 3. Hypergonadotropic Hypogonadism

An insufficient or nonexistent function of the testicles is the primary disturbance in hypergonadotropic hypogonadism. Because there is no negative feedback from testicular testosterone, estradiol, or inhibin B, gonadotropin levels are adequately raised. Spermatogenesis is hampered if the body does not produce enough androgens. As well as testicular shrinkage and fibrosis, these men often have significantly diminished germ cell numbers, which contributes to an unusually reduced rate of spermatogenesis in the testicles. It is possible to develop hypergonadotropic hypogonadism due to genetic or acquired causes. Although exogenous testosterone therapy can be used to treat males suffering with hypergonadotropic hypogonadism who do not wish to become pregnant, it should be avoided in men who are attempting to conceive. A lesser amount of research has been done on the treatment of males who are attempting to conceive. 

Infertile men with hypergonadotropic hypogonadism are treated with medical therapies alone or in combination with ARTs. Gonadotropins, selective estrogen receptor modulators (SERMs), aromatase inhibitors (AIs), and their combinations are among the available treatment options [43]. Men suffering from Klinefelter syndrome (KS) have been recommended to use aromatase inhibitors [15]. Treatment of a small group of patients suffering from KS who were using estrogen-blocking drugs revealed that their hormone levels improved, albeit no information was provided about the menstrual characteristics of this subset. Furthermore, testolactone medication was found to be more effective than anastrozole for these patients when it came to hormone levels [44]. When surgical sperm extraction follows adjuvant medical therapy in males with KS, it is crucial to point out the additional potential benefit that may be gained. In some cases, surgical sperm extraction alone has been shown to result in effective retrieval in up to 50% of cases [23,45]. In a retrospective study involving a group of 68 KS males of reproductive age, 56 were treated for reduced levels of testosterone (300 ng/dL) with a combination of pharmacological medications (aromatase inhibitors, clomiphene, and hCG) before undergoing microdissection TESE [45]. One hundred and sixty-six (56%) of the men who underwent medical therapy prior to TESE were treated with anastrozole alone, one hundred and twelve were treated with anastrozole and weekly hCG, nine were treated with anastrozole and weekly hCG, four were treated with hCG only, and clomiphene citrate was administered to three patients. In terms of successful sperm extraction, there was no significant difference between specific agents; however, when patients reacted to medical therapy by having posttreatment testosterone levels more than 250 ng/dL, these medical regimens resulted in enhanced sperm retrieval. More specifically, effective sperm extraction was observed in 77% of men with posttreatment testosterone > 250 ng/dL, compared to 55% of men with posttreatment testosterone 250 ng/dL [45]. When LH levels grow above normal throughout puberty, testosterone supplementation is most commonly used to treat the problem. Research projects now underway may contribute to the development of evidence-based guidelines for androgen supplementation timing, despite a paucity of available data. Because the trial was neither randomized and blinded, the generalizability of the findings is uncertain [46]. A retrospective study reported that infants with KS who received testosterone treatment had superior cognitive development at 3 years and 6 years old. A randomized controlled experiment on KS in early adolescence is now enrolling participants with the goal of examining the psychosocial impact of topical testosterone [47] in this population. Studies now underway may contribute to the development of evidence-based guidelines for the timing of androgen supplementation, despite a paucity of available information. Since KS testing may soon be integrated into standard prenatal care in the near future, the rate of KS diagnoses is expected to increase significantly in the coming years [48]. It is expected that this adjustment will result in an increase in the rate of diagnosis of up to five times the existing rate. Therefore, a higher rate of diagnosis may encourage the research community to devote more time and resources to studying patient-centered KS health and treatment outcomes.

## 4. Androgen Excess

It has been shown that testosterone acts as a negative feedback inhibitor on the hypothalamic secretion of GnRH in the testis. This is an indirect impact that is considered to be caused by the aromatization of testosterone into estradiol. Excess testosterone in the bloodstream can act in this manner, suppressing this axis and resulting in the suppression of spermatogenesis. Excess testosterone can be caused by either exogenous testosterone administration or endogenous testosterone synthesis. Inadvertent testosterone overproduction can occur as a result of therapeutic treatment, but testosterone overproduction can also occur as a result of the illicit use of anabolic steroids. In general, exogenous androgens reduce gonadotropin release, resulting in decreased intratesticular testosterone levels and decreased spermatogenesis, regardless of the underlying etiology. The presence of normal-to-high serum testosterone levels in conjunction with reduced gonadotropins suggests the presence of the condition. The first step in treating a male who has been diagnosed with androgen excess is to identify and eliminate the exogenous source of the extra androgen. The return of spermatogenesis normally happens within four months; however, it has been reported that it might take up to three years in some situations [16,49]. If the sperm parameters do not improve sufficiently or do not improve quickly, some evidence shows that GT may be advantageous in increasing intratesticular testosterone levels [17]; however, this has not been proven. If a patient’s response to treatment remains unsatisfactory following a trial of gonadotropin medication, limited evidence suggests that clomiphene may be used to reestablish the hypothalamic–pituitary–gonadal (HPG) axis [50]. Anabolic androgenic steroids (AAS) usage has not been a major topic of discussion in mainstream medicine until recently, when a new study on young men in the United States revealed the negative health impact of AAS. A retrospective intervention on more than 6000 patients suggested that steroid abuse contributed to the etiology in more than one-third of hypogonadism patients. Moreover, about one-fifth of men treated for symptomatic hypogonadism reported previous use of anabolic androgenic steroids [51]. As the prevalence of steroid addiction continues to rise, an increasing number of psychiatrists are recognizing anabolic androgenic steroid dependency as a distinct diagnostic entity [52]. The ability to counsel patients suffering from anabolic steroid-associated hypogonadism and to understand their motivation for use is critical for both preventing future use and recognizing other diseases the patient may be suffering from, such as primary hypogonadism, that the provider can safely treat medically. Endogenous androgen synthesis can also result in an increase in androgen levels. While congenital adrenal hyperplasia is the most prevalent endogenous cause, other possibilities include functional tumors (adrenal or testicular) and androgen insensitivity disorders [12,30]. However, despite the fact that congenital adrenal hyperplasia is most usually discussed in the context of female fertility, there have been numerous studies associating the disease to lower male fertility [53,54]. When it came to attempting pregnancy, just two-thirds of males with congenital adrenal hyperplasia were successful, according to one of these studies [55]. In terms of treatment, a variety of approaches have been examined and found to be effective, including the use of hCG in conjunction with FSH, clomiphene citrate, and intracytoplasmic sperm injection [56,57].

## 5. Estrogen Excess

It has already been mentioned that testosterone’s capacity to limit GnRH release at the hypothalamus is mediated by the hormone’s conversion into estrogen. A primary excess of estrogens can work in a similar manner to have an inhibitory effect on the HPG axis, resulting in lower fertility in both men and women. As with females, testosterone and estrogen are created in the testicles, but the main source of estrogen in males comes from the peripheral aromatization of testosterone by the enzyme aromatase, which can be found in adipose tissue. Because of the increasing incidence of obesity in our society, more men are at risk of developing estrogen excess. Many doctors believe that the ratio of testosterone to estradiol (T:E2), in particular, is a crucial indicator of estrogen excess, with a goal ratio greater than 10:1 being sought by many. Pavlovich et al. [24], when they analyzed a cohort of infertile men, discovered that their T:E2 ratios were much lower than those of the fertile control group (6.6 versus 14.5). Inhibitors of the aromatase enzyme are used to treat women who have a relative estrogen excess. Aromatase inhibitors are classified into two categories: steroidal drugs (for example, testolactone) and nonsteroidal medicines (for example, ethinyl estradiol) (e.g., anastrozole). Both have been demonstrated to be effective in the treatment of infertile males who have low T:E2 ratios. Testolactone, 50–100 mg twice day, was used in the Pavlovich research to treat 63 men who were suffering from male factor infertility and low T:E2 ratios. Increasing the T:E2 ratio and enhancing sperm quality, as measured by concentrations and motility, were found to be successful treatment strategies [40]. Raman and Schlegel conducted a trial in which they treated 140 infertile men with aberrant T:E2 ratios with either testolactone (100–200 mg daily) or anastrozole (100–200 mg daily). Both treatment arms demonstrated an improvement in the T:E2 ratio, as well as increased sperm concentration and motility in both cases. Aside from KS, where testolactone was found to be superior in treating the aberrant T:E2 ratios, the study did not find any statistically significant differences between the two types of aromatase inhibitors when it came to hormonal profile or semen analysis. These trials, taken together, demonstrate that aromatase inhibitors have a clear function in the treatment of infertile males with aberrant T:E2 ratios. This therapy technique may be particularly beneficial in the treatment of obese people [58].

## 6. Hypothyroidism

**Figure 1:**
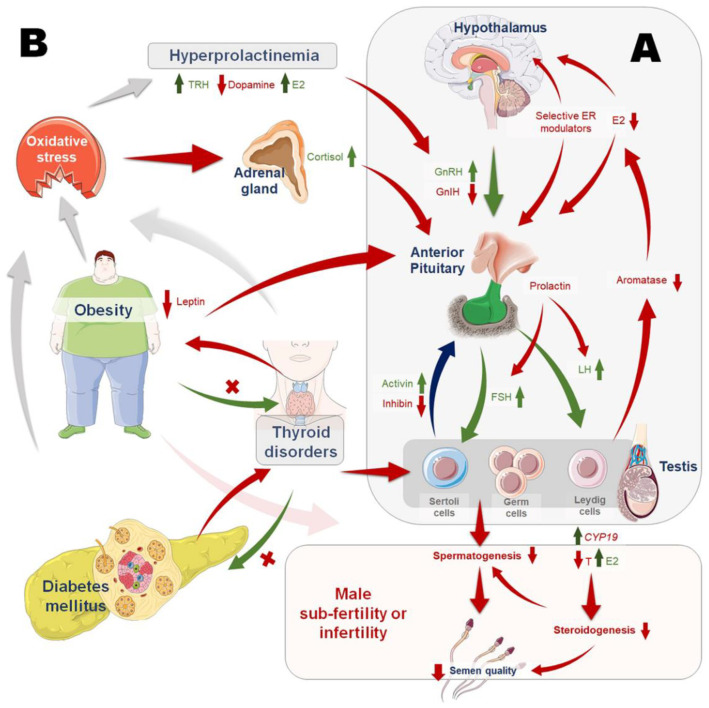
Endocrinopathies and male reproduction. (**A**) Neuroendocrine regulation by hypothalamic–pituitary–gonadal (HPG) axis maintains the normal secretion and functions of reproductive hormones. Gonadotropin-releasing hormone (GnRH) is synthesized by the hypothalamus, which stimulates the anterior pituitary to secrete the gonadotropins, luteinizing hormone (LH) and follicle-stimulating hormone (FSH). Whereas, gonadotropin-inhibitory hormone (GnIH) inhibits the anterior pituitary gonadotropin synthesis and release. In Leydig cells, LH acts to aid steroidogenesis. FSH acts on the Sertoli cells, supporting spermatogenesis. Sertoli cells secrete activin and inhibin among other substances, which mediate positive and negative feedback on the HPG axis, respectively. (**B**) Hormonal disturbances owing to endocrinopathies can impair hormonal crosstalk, thereby disrupting essential male reproductive functions. Upregulation of aromatase CYP19 (Cytochrome P450 Family 19) gene leads to a higher conversion rate of testosterone to estrogen, inducing estrogen excess, which in turn inhibits the HPG axis. Hyperprolactinemia is characterized by high serum prolactin levels that impede GnRH release from the hypothalamus, reducing gonadotropin secretion and perhaps inhibiting gonadotropin actions on the gonads. Endocrinopathies including obesity, thyroid hormone imbalance, and diabetes mellitus disrupt the intricate metabolic balance, elicit various metabolic hormones and inflammatory mediators, and may induce oxidative stress, all of which adversely affect the normal endocrine crosstalk regulating male reproductive functions.

Thyroid hormones are necessary for the development and functioning of tissues as well as to maintain normal body metabolism [59,60,61]. However, there have only been a few human investigations examining the link of hypothyroidism with male infertility/subfertility [62,63,64,65] (Figure 1). Although it has been established long before that adult-age hypothyroidism leads to reduced sexual desire [66], the exact association of hypothyroidism with infertility has only lately been explored. While hyperthyroidism is associated with elevated sex-hormone-binding globulin (SHBG) levels, human studies have reported that hypothyroidism leads to a reduction in levels of SHBG and total serum testosterone [65,67]. Hypothyroidism, in contrast to hyperthyroidism, is reportedly associated with lower free testosterone levels [68]. When it comes to exogenous GnRH, hyperthyroid individuals have an increased response, which can be ascertained by a diminished response in hypothyroid individuals [62]. In a few hypothyroid cases, men also have shown hypothyroidism-mediated diminutive basal levels of LH and FSH [69]. Having this condition in a prepubertal male for an extended length of time would result in reduced gonadotropin-mediated Leydig and Sertoli cells functions, which could result in a reduction in the maturation of the sperm itself. Although the number of cells in the testis would increase, the number of mature cells would decrease as a result of this procedure. This may serve as a plausible reason for the increased testicular size observed in some hypothyroid patients, and it has been shown to be connected with a decrease in mature germ cells in the seminiferous tubules [70].

In order to better understand the link of hypothyroidism with male fertility, extensive research was conducted on rats. In comparison to the control rat group, induced-hypothyroidism rat models showed lighter testes, lesser number of testicular germ cells, tinier and fewer seminiferous tubules, and deteriorated sperm parameters [63,64,71]. Long before the discovery of the thyroid hormone, hypothyroidism was related with decreased libido and erectile dysfunction [72]. Moreover, a concurrent study had projected that higher thyroxine (T4) levels associate significantly with enhanced sperm concentrations [73]. Besides sperm concentration, hypothyroidism has also been shown to decrease the percentage of sperm of normal morphology, disrupt sperm motility, as well as reduce the semen volume. About 76% of individuals had normal morphology once their hypothyroidism was corrected, according to one study [74]. However, the largest study to date found significant changes in sperm morphology between 23 hypothyroid and 15 euthyroid males [74], despite the fact that no studies have yet established a difference in sperm motility between hypothyroid and euthyroid men. It has also been shown that primary hypothyroidism in prepubertal males is associated with histologic abnormalities of the testicular cells, which is consistent with the theory that low LH and FSH levels in hypothyroid males result in an abnormal number of immature germ cells in the seminiferous tubules [70]. Just as has been demonstrated in the case of hyperthyroidism, treatment of the underlying thyroid hormone imbalance can enhance semen parameters [67,74]. A relative dearth of information exists regarding hypothyroidism and the characteristics of the male reproductive system. Despite this, the findings of these studies imply that there is a relationship between thyroid function and sperm production (Table 2; [63,64,68,69,71,73,75,76,77,78,79,80,81,82,83,84,85,86]).

Hypothyroidism may also result in hyperprolactinemia due to elevated levels of thyrotropin-releasing hormone (TRH), leading to infertility.

## 7. Hyperthyroidism

As previously mentioned, the exact involvement of thyroid hormones in the spermatogenesis is only partially understood [59]. Hyperthyroidism, on the other hand, appears to have a negative impact on sperm parameters [78] (Figure 1). Compared to healthy controls, individuals with hyperthyroidism have been shown to possess greater levels of SHBG and LH, but lower free testosterone levels [78]. Patients with hyperthyroidism have been found to have significantly compromised sperm parameters, including low motility, low ejaculate volume, low sperm concentration, and aberrant sperm morphology. After achieving an euthyroid condition, the investigators reported that semen tests performed 7 to 19 months following an euthyroid state showed restoration of 85% of seminal abnormalities. As per another study, hyperthyroidism was shown to have various adverse effects on sperm parameters [87], which were restored on achieving an euthyroid level with medical thyroid ablation. As with hypothyroidism, there is a paucity of information on the relationship between hyperthyroidism and spermatogenesis [66,78]. The available evidence, on the other hand, appears to indicate that hyperthyroidism can have adverse impact on sperm parameters.

There has been extensive research into the effect of thyroid function on fertility in a variety of animal models, with the majority of studies concluding that when thyroid hormone levels deviate from normal ranges, the effect on fertility and libido is negative [66,71,88]. Mechanisms differ significantly between the various species under investigation, making it difficult to reach a consensus on specific claims. Increased levels of SHBG in humans [89] are a well-known feature of hyperthyroidism, which results in elevated levels of serum testosterone. It appears that thyrotoxicosis has no effect on the biologically available form of testosterone, known as free testosterone [90], leaving the clinical consequences of the condition unclear. A similar pattern has been observed in many men with thyrotoxicosis, with elevated levels of circulating E2 possibly due to increased binding of E2 to SHBG [84]. In some men, an increase in the amount of SHBG-bound estrogen is accompanied by an increase in the rate at which estrogens are produced [85]. This is consistent with the stigmata associated with increased estrogen exposure that frequently accompanies hyperthyroidism in men, such as gynecomastia, spider angiomas, and decreased libido [91]. When compared to euthyroid controls, the levels of gonadotropins in men with hyperthyroidism are usually normal. However, some studies have discovered that the LH and FSH responses to GnRH are exaggerated in hyperthyroid patients compared to euthyroid controls [91]. These studies would appear to support the notion that thyroid hormone levels are related to gonadotropin sensitivity (or sensitivity to estrogen). Others have observed an increase in basal LH and FSH levels, as well as a hyper-responsiveness to exogenous GnRH in hyperthyroid patients, which has been linked to the condition [86].

## 8. Hyperprolactinemia

Hyperprolactinemia, defined as an excess of the hormone prolactin, is among the major endocrinopathies related to male infertility [25] (Figure 1). The diagnosis is rather straightforward, as hyperprolactinemia may be found with routine serum tests; however, determining the origin of the condition can be difficult. Hyperprolactinemia can arise as a result of hypothyroidism, liver illness, stress, and the use of certain drugs (such as phenothiazines and tricyclic antidepressants), as well as in the presence of functional pituitary adenomas [25,26]. The symptoms of excess prolactin may be asymptomatic in some cases or lead to hypoandrogenic state or galactorrhea, while reduced libido and erectile dysfunction are reported in the other cases [26,27]. Hyperprolactinemia can cause male infertility due to its inhibitory effects on hypothalamus [28]. As a result, the hypothalamus is unable to secrete gonadotropins, which in turn affects testosterone production and spermatogenesis. Prolactin levels that are too high are associated with a decreased ability to produce testosterone [28]. Because of the numerous impacts on the HPG axis, a patient may present with a variety of symptoms, including diminished sexual desire, erectile dysfunctions, and reduced semen quality [25]. Once hyperprolactinemia has been diagnosed, the practitioner should order an MRI scan of the pituitary gland to rule out any other potential causes. If a prolactinoma is discovered, it can be classified according to its dimension and form. The most important distinction is between microadenomas, which are lesions smaller than 10 mm in diameter, and macroadenomas, which are lesions larger than 10 mm in diameter. The medical treatment for prolactinoma is focused on inhibiting the release of prolactin with the use of dopamine agonists if the tumor is found to be present, which include, pergolide, cabergoline, bromocriptine, and quinagolide, with cabergoline and bromocriptine being the most well-characterized agents [25,92]. These agonists take advantage of dopamine’s inherent suppression of prolactin release to achieve their effects. In certain cases, this can really result in the tumor shrinking, albeit the process usually takes months to complete. Nausea, vomiting, and postural hypotension are among side effects that can occur after using dopamine agonists. However, despite the fact that inhibition of excess secretion of prolactin prevents disruption of the HPG axis, few studies have examined the effects of dopamine agonists on reproductive functions. Bromocriptine was used in a 1974 trial to treat men with functional prolactinomas and hypogonadism, and the results revealed no increase in sperm motility [93]. On the other hand, a study compared cabergoline and bromocriptine in the same cohort of patients, and both treatment regimens showed significant improvements in sperm quantity, motility, rapid advancement, and morphology in a period of six months [94]. In a later study conducted at the same institution, seminal fluid parameters were compared between men who had prolactinomas and men who did not. According to a study conducted on healthy control males, after 2 years of treatment with cabergoline (starting dose 0.5 mg weekly, gradually titrated to PRL levels), the majority of men had regained testicular functions in comparison to the healthy control.

When bromocriptine and cabergoline are compared, it is observed that cabergoline has higher effectiveness at normalizing levels of prolactin and regressing tumor load [95]. Furthermore, when comparing cabergoline to bromocriptine, a higher percentage of individuals demonstrate a clinical response to cabergoline. Finally, compared to bromocriptine, cabergoline has a considerably better rate permanent remission rate and fewer side effects [95]. All things considered, cabergoline is frequently the initial treatment option for males with prolactinomas after other options are exhausted. In many situations, treatment of prolactinomas with dopamine agonists is beneficial; nonetheless, a considerable proportion of men may still remain persistently hypogonadotropic despite receiving treatment. The use of clomiphene citrate, according to a study, may be a successful therapy option for these men. Hypogonadal men treated with clomiphene (50 mg per day for 3 months) had increased levels of testosterone as well as improved sperm motility [96]. Prolactinomas can be treated with ablative therapies such as radiation therapy or transsphenoidal excision, which are both effective. Ablative therapy is usually reserved for patients who have failed to respond to medical treatment. Ablative treatments work by removing the prolactin source and, as a result, the suppression of GnRH secretion that is occurring. It is still vital to monitor the patient’s gonadotropin levels after treatment since additional intervention with exogenous gonadotropins may be required to maximize therapeutic benefit. Treatments for male infertility have generally relied on empirical ways to increase spermatogenesis. However, this is changing. Over the past two decades, researchers have obtained a better understanding of the biology of male infertility as well as the outcomes related with empirical fertility treatment. It has been proposed that medical agents be used in a more targeted and directed manner as a result of this knowledge. This has led to a decrease in the usage of ‘empiric therapy’ compared to what was used two decades ago. Various therapies for male infertility are utilized to improve the hormone milieu, which in turn helps to maximize spermatogenesis in the male partner. Exactly this has been the primary topic of this chapter. Numerous additional medicinal treatments, on the other hand, are routinely utilized to treat a variety of different particular pathophysiologic disorders that contribute to male subfertility. Sympathetic agonists, antimicrobial, and anti-inflammatory pharmaceuticals are some of the agents in this class. There are clear indications for the use of each of these pharmacological classes in specific male infertility cases. One key point that has been clearly established in the literature over the last few years is that empirical medical therapy is generally of low utility and benefit in the treatment of infertility in men, as has been demonstrated in numerous studies. Despite the fact that randomized, placebo-controlled, double-blinded interventions are time- and money-consuming, they continue to be the gold standard for determining whether or not a medical treatment is successful. In past few years, more than one agent has failed in that regard, yet this is positive progress. While the number of medical treatments for male infertility is minimal, this should drive us to further research the pathophysiological mechanisms that lead to male infertility. This improved perception of the underlying issues that contribute to male infertility will allow us to design further, more effective medical treatments for male infertility.

## 9. Insulin Disorders and Diabetes Mellitus

Studies show that diabetes has negative impacts on both male and female reproduction [19,97,98,99], and that the consequences of this are reflected in an increased prevalence of infertility [100,101]. According to the American Diabetes Association, about 90% of diabetes cases are accompanied by changes in their reproductive functions, diminished libido, and infertility or subfertility [102]. Moreover, men with diabetes have been shown to be more susceptible to a variety of sexual issues, though both growing physical illnesses and a deteriorating psychological reaction play a role [103], as previously mentioned. Several studies have researched and reported on the various diseases that diabetic males typically suffer from, as well as the resulting reproductive problems that might result from these conditions. 

Spermatozoa can generate energy by both glycolysis and oxidative phosphorylation. They are capable of producing energy both from exogenous hexoses (such as glucose, mannose, and fructose), as well as other substrates (such as amino acids, citrate, lactate, and lipids). Despite the fact that spermatozoa are able to produce their own insulin, these cells remain sensitive to hormonal alterations [104]. Consequently, in diabetes, insulin insufficiency or insulin sensitivity affects the endocrine route (negative feedback loop), resulting in reduced male reproductive function as a result (Figure 1).

Several animal investigations on induced hyperglycemia demonstrated some negative effects on male reproductive function, which were associated with impaired endocrine control. Additional effects of diabetes include reduced vacuolization in the Sertoli cells [26], reduced spermatogenesis [19,20], decreased fertility [99], changes in the morphology of the epididymis [21], reduced levels of gonadotropins and serum testosterone [105], and diminished count of germ cells, Leydig, and Sertoli cells [99]. These impacts of diabetes mellitus upon spermatogenesis have been thus proven via both animal and human studies.

A further study by Ballester et al. [22] found a reduction in Leydig cells count and functions in diabetic mice models induced with streptozocin (STZ). The drop in Leydig cells count was associated with a reduced serum LH, which may partially explain the stimulating role of LH on the Leydig cells in the laboratory setting. Moreover, it was discovered that LH is a mediator of Leydig cell formation, which involves signaling processes that involve insulin and insulin-like growth factor 1 [106,107]. However, tyrosine phosphorylation was completely inhibited, and expressions of androgen receptors, GLUT-3 receptors, as well as the insulin-like growth factor 1 receptors were all downregulated [108]. The altered cell function was also observed in the absence of tyrosine phosphorylation. In addition to these findings, several other animal studies [109,110,111] have looked into the effect of diabetes on male fertility and come to similar conclusions. Additionally, diabetes reportedly affects the spermatogenic cycle by impeding the FSH actions on the Sertoli cells [22,112]. Insulin insufficiency in type I diabetes does not seem to affect spermatogenesis by directly affecting the seminiferous epithelium, but rather through a shift in serum FSH levels. With a reduction in FSH levels, a decrease in tubular FSH receptors is observed in type I diabetes caused by STZ. This results in a reduced response to FSH stimulation by the epithelium of the seminiferous tubules. Because of this, diabetes interferes with spermatogenesis by interfering with insulin’s regulating influence on serum FSH levels [22,112].

A similar finding has been made about the role of glucose role in spermatogenesis and the acrosome reaction (AR) [113], where a medium without glucose hindered the spontaneous AR, which was quickly recovered after the addition of glucose to the media. GLUTs are responsible for transporting these substrates into the cell [114]. GLUTs are specialized transporters catalyzing the passive glucose diffusion into cells. A total of 14 members make up the GLUT family, which can be split into three groups based on the sequence similarities between them [115].

It is known that GLUT8 is a member of the class 3 transporters and that it is expressed preferentially in the testis [116,117]. It was discovered in mature human spermatozoa, according to research on the expression of the GLUT8 gene [118], that the gene is expressed in the acrosome and midpiece region. The acrosome and midpiece areas of mature spermatozoa from mice were similarly reported to contain the molecule [119]. As previously stated, some studies have discovered GLUT8 in developing spermatocytes of the stage 1 type, but none have discovered it in mature spermatozoa [116]. The glucose that is carried into the cell is turned into energy, which is required for spermatogenesis and cell motility to occur. Reduced sperm motility and poor fertilization were seen as a result of the disruption of GLUT8 function mediated by lower insulin levels [120]. As previously stated, diabetics have a decreased gonadotropin response to GnRH [106], which could explain this phenomenon.

## 10. Obesity and Endocrine Disruption

The mechanisms by which obesity is linked to male infertility remain largely unidentified [121,122]. The obesity-associated impairment of the HPG axis regulations of testicular function may be the most acceptable mechanism to explain this phenomenon [123]. The pituitary gonadotropins are controlled by the pulsatile hypothalamic GnRH release. LH acts on Leydig cells, primarily regulating steroidogenesis, while FSH acts on Sertoli cells, primarily regulating the process of spermatogenesis [124]. Overweight or obese men have larger and higher number of adipocytes, which generate more adipokines and metabolic hormones, increasing the levels of inflammatory mediators in the circulation [125]. Adipose tissue-secreted molecules modulate the intricate regulation of the HPG axis, which may help to understand the mechanism of how the obesogenic attributes lead to male subfertility. Studies have reported that the typical obesity-related parameters, such as the body mass index (BMI), total body fat, abdominal fat, and subcutaneous fat in men, correlate to lower testosterone levels and increased concentration of estrogen [126,127]. One possible explanation is that in obese men, activities of the estrogen-metabolizing enzyme, aromatase cytochrome P450, are exceedingly high. This enzyme is expressed in excess by white adipocytes relative to that by Leydig cells. Aromatases convert androgens to estrogens, and thus obese men have a high estrogen level [128]. Male reproductive functions, including spermatogenesis and other androgen-dependent functions, are affected by such changes in sex hormones. Estrogen, being more biologically active than testosterone, has the potential to cause significant downstream effects with even a small increase in its plasma levels, resulting in the disruption of testicular functions [129]. In fact, a total estrogen reduction in the testis interferes with normal steroidogenesis and spermatogenesis [130]. Increased estrogen levels in obese men are thought to be caused by a negative feedback that inhibits the GnRH released in pulses and thereby also hinder LH and FSH release, according to the hypothalamic ERs expressions [131]. This mechanism ultimately results in a deficiency of gonadotropins, which in turn results in insufficient androgen synthesis and spermatogenesis. Inhibin B, a growth-like protein released by Sertoli cells, also functions as a feedback inhibitor of FSH synthesis. It also stimulates the Leydig cells to produce testosterone. A high estrogen level or another mechanism could be responsible for obesity-induced reduction of inhibin B production in obese males [132].

Obesity causes a variety of bodily disorders that have a significant impact on the physiological hormonal milieu [124]. Due to excess white fat accumulation in obese men, estrogen levels rise, and adipose tissue hormones surge, which has an impact on the hormones that play a role in steroidogenesis and spermatogenesis. As previously discussed, an increase in estrogen levels is caused by an enhanced aromatase activity, which converts testosterone into estrogen. In the human body, adipose tissue serves as a major energy source as well as an endocrine gland by secreting hormones. It is possible for these tissues to synthesize a variety of bioactive substances, such as adipokines, which stimulate chronic low-grade inflammatory responses and interact with numerous metabolic pathways [133]. The accumulation of excess fat results in the release of free fatty acids into the circulation, which is a critical factor in the regulation of insulin sensitivity. However, adipokines must be maintained at physiological levels to ascertain normal metabolic functions [134]. 

Obesity stimulates the release of hormones from adipose tissue, including leptin, ghrelin, orexins, obestatin, adiponectin, and other metabolic hormones, which possess unique roles in reproductive functions [8,135,136,137,138,139]. Leptin regulates the satiety center and body weight primarily through three hypothalamic leptin-sensitive neurons: neuropeptide Y, γ-aminobutyric acid (aminobutyric acid), and proopiomelanocortin neurons [140]. Despite the fact that leptin has the ability of crossing the blood–brain barrier, it has inhibitory effects on neuropeptide Y and gamma-aminobutyric acid neurons, which have actions upon the proopiomelanocortin neurons and brings about the satiety sensation while increasing the amount of energy expended [125]. As a result, leptin, a regulatory adipose tissue hormone, maintains a healthy balance between food intake and energy expenditure via its impacts on hypothalamic control [135]. According to reports, leptin has important roles in metabolism as well as in neuroendocrine regulations. Aside from its roles in glucose metabolism, leptin is involved in the endocrine control of male reproductive maturation and functions. It has been demonstrated on obese mice lacking leptin gene that the absence of functional leptin may lead to suppressed gonadotropin secretion, which results in infertility, whereas exogenous leptin treatment restores fertility in the obese mice [141]. When laboratory rats were administered with anti-leptin antibodies over an extended period of time, LH secretion and testicular functioning were inhibited. Leptin also bears a regulatory role in spermatogenesis, as evidenced by the fact that leptin-deficient mice had impaired spermatogenesis as well as increased pro-apoptotic genes expression levels in the testis, resulting in the induction of germ cell apoptosis [142]. Only a few reports have been published that oppose the beneficial impacts of leptin on male reproduction, demonstrating its inhibiting effects on testicular functions when administered in very high doses [143]. Leptin induces reactive oxygen species (ROS) generation in human endothelial cells by elevating the rate of mitochondrial oxidation of fatty acids [144,145]. Leptin has been shown to upregulate the HPG axis via induction of GnRH, FSH, and LH, among other hormones [146]. It has the ability to exert a direct influence on the gonads because its receptor isoforms are found in high concentrations in the testis [146]. The serum adiponectin concentrations are shown to be inversely related to those of testosterone [147] and ROS [148]. 

It is possible that leptin, through its influence on kisspeptin, can control hypothalamic GnRH secretion. Almost everyone agrees that kisspeptin is important in reproductive endocrine regulations. Kisspeptin is found in the arcuate nucleus of the hypothalamus and may act as a connection between metabolism and reproduction [149]. Kisspeptin reportedly suppresses lipogenesis while simultaneously inducing lipolysis [150]. Metabolic syndromes, such as obesity, are characterized by decreased hypothalamic and adipose tissue kisspeptin mRNA (KISS1) expressions [149]. Because kisspeptin increases the hypothalamic GnRH release in pulses, obesity-mediated inhibition of kisspeptin may lead to hypothalamic hypogonadism [149,150]. Another developing adipose tissue hormone, orexins (hypocretins), apparently increases testosterone synthesis by increasing activity of steroidogenic enzymes in the Leydig cells [151]. Orexins appear to be protective against oxidative cell damage as well [152,153].

Obese males have been found to have a significantly higher secretion of resistin from their adipocytes, according to several studies. Resistin has the potential to cause insulin resistance (IR) in obese males, resulting in type 2 diabetes [154,155]. According to the ‘The Endocrine Society Clinical Practice Guidelines (2010)’, men suffering from type 2 diabetes should undergo tests for low levels of testosterone [98]. Type 2 diabetes is accompanied by IR in obese men, which may lead to secondary hypogonadism. Pro-inflammatory cytokines (interleukin 6 and tumor necrosis factor-alpha) act on the HPG axis, aggravating the disease [128,156,157]. Obese men’s elevated insulin levels may also diminish SHBG levels, resulting in lower testosterone functions than are required for optimal spermatogenesis. Since compensating low SHBG levels for low testosterone levels in obesity has failed, it is possible that IR has a direct impact on Leydig cell production of testosterone [128,158]. Ghrelin is called the ‘hunger hormone’ since it causes people to feel hungry. According to some research, ghrelin is a neuropeptide that is secreted by gastrointestinal ghrelinergic cells and is reportedly associated with lower serum testosterone levels in obese men [159,160,161]. Ghrelin receptors can be located in the testicles, and they play an important part in the process of steroidogenesis. The direct impact of ghrelin on spermatogenesis, on the other hand, is still up for debate [159]. Ghrelin may accelerate ROS and induce oxidative stress, which can interfere with normal testicular activities [162]. The hormone adiponectin has a diametrically opposed relationship to fat and IR. It predominantly impacts upon the skeletal muscle, liver, and the endothelial cells that line the inside of blood vessels. It has the ability to promote nitric oxide synthesis, which aids in the process of angiogenesis [163]. It helps in managing obesity-associated nonalcoholic steatohepatitis, a condition characterized by redness, and accumulation of fat and gristly tissues in the liver, among other symptoms [164]. Vaspin functions in the progression of obesity and metabolic dysfunctions, and has a role in the development of diabetes and IR. Body fat percentage, BMI, and blood glucose levels are all substantially correlated with the visceral expression of vaspin mRNA in humans. It follows a sex-based direction, and the occurrence of the disease is significantly higher in women compared with men [165]. Apeline [166], fatty acid-binding and acylation-stimulating peptides, visfatin [156], omentin [167], chemerin [168], irisin [169], and plasminogen activator inhibitor-1 are some of the other adipokines that have been discovered recently.

All of the obesity-related hormones that have been discovered to date, including those mentioned above, are able to only partially reveal the complex mechanisms by which obesity paves ways to male infertility. Future in-depth studies are required to identify an adipokine that is ‘derived from fat’ and that aids us in our ‘fight against fat’. Obesity has emerged as a major public health concern in both developed and developing countries in the twenty-first century. Socioeconomic status changes, unhealthy eating habits, a stressful lifestyle, and a lack of physical activities all contribute to developing obesity and related disorders [170]. 

Despite their dynamic nature, the seminiferous tubules maintain an equilibrium between cell growth and death [171]. Following the first spermatogenic wave, there is a period of differentiation of germ cells, which is governed by complex hormonal signals. The B-cell lymphoma-xL (Bcl-xL)- and Bcl-2-associated X protein (Bax) systems direct cells to undergo apoptosis if cell differentiation in this phase exceeds the physiological limit [172,173]. Specific physiological or pathological conditions might cause spermatogonial apoptosis. In obese people, spermatozoa from artificial insemination have been found to have a high rate of apoptosis. Obesity-induced germ cell death is responsible for the majority cases of male subfertility and infertility [174]. The traditional Bax and Bcl-2 balance regulates spermatogonial apoptosis. Obesity alters the Bcl-2/Bax ratio in the testis, boosting Bax while decreasing Bcl-2 expression. These changes could activate downstream apoptotic signaling caspases, particularly caspase 3 in spermatogonia [123]. Furthermore, obesity-related hyperlipidemia and lipid metabolic disorders cause the endoplasmic reticulum to promote spermatogenic cell death by increasing GRP78 mRNA and protein expression [175,176].

Human sperm quality is a well-established male fertility predictor, which is on the decline around the world [4,5,177,178]. It has been widely observed that obesity and overweight, as well as the associated allostatic load, are linked to a higher occurrence of azoospermia and oligozoospermia in males and females, respectively [179]. Proper control and disciplined weight loss resulted in a significant increase in testosterone concentrations and improvements in sperm functions [180]. The most validated and conventional male fertility parameter is semen quality, which mainly includes sperm count, sperm morphology, sperm motility, and semen volume. The properties of seminal fluid in males are influenced by their general reproductive health as well as external signals. Even a slight deviation from homeostatic conditions can result in a decrease in the parameters of the spermatozoa. Trauma, systemic illnesses, an unhealthy lifestyle, low nutritional status, environmental stress, and metabolic abnormalities, such as obesity, can all have a negative impact on the quality of sperm and the ability to reproduce [181]. The link between a high BMI and poor steroidogenesis and spermatogenesis, as well as deteriorated semen quality, has been established, although further research is needed to confirm this association in more detail [158].

In comparison to normal-weight men, obese men have been shown to have three times higher possibility of sperm counts less than 20 × 10^6^/mL. ‘Oligozoospermia’ is the name used to describe this condition of decreasing sperm count [129]. Chavarro et al. [182] showed in their comparison of overweight and obese males that those with a higher BMI (>25 kg/m^2^) had a reduced total sperm count compared to those of normal weight. A rise in body mass index (BMI) is associated with a decrease in the volume of semen ejaculated. Additionally, a broad-spectrum investigation involving 1558 Danish military males found a negative relationship between higher BMI and total sperm count and concentration (both of which were low) [183]. Obesity has also been reported to alter sperm morphology and motility, while the exact process is still being investigated [184]. These data have been used in a number of studies to imply that obesity has an adverse effect on male reproductive health [127,185].

## 11. Endocrinopathies, Oxidative Status of Male Reproduction

Due to the disruption of the balance between oxidants and antioxidants in the male reproductive system, the majority of these hormonal abnormalities might result in the generation of ROS [71,103,144]. These ROS interfere with the crosstalk between distinct endocrine axes, which is detrimental to male reproductive functions. Increased production of ROS results in lipid peroxidation of Leydig cells and developing testicular cells, lipoprotein degradation, protein aggregation, DNA fragmentation, and enzyme inhibition. [186,187]. Testicular OS results in a decrease in testicular testosterone production as a result of damage to the Leydig cells or endocrine tissues such as the anterior pituitary [188,189]. The natural steroidogenesis process generates ROS, mostly from mitochondrial respiration and steroidogenic cytochrome P450 enzyme catalysis [190]. These ROS damage spermatozoa mitochondrial membranes and also inhibit steroidogenesis. [191]. OS is connected with an increase in the percentage of immature spermatozoa through an indirect effect on the production of male hormones [192,193,194,195]. A favorable link has been demonstrated between PRL and free T4 (fT4) with total antioxidant capacity (TAC), but not with gonadotropins or gonadal steroids. It has also been observed that systemic hormones may modulate seminal TAC [196].

It is undeniable that certain hormones, including testosterone and melatonin, may act as antioxidants, protecting sperm and other testicular cells from the damage caused by ROS [197,198]. Other steroidogenic pathway metabolites, such as DHEA, have been shown to increase the level of cellular antioxidants, albeit the exact process is still unexplained [199]. Researchers have discovered a direct communication between testosterone and antioxidants such as selenium and/or coenzyme Q10 (CoQ10), as well as an indirect communication between testosterone and zinc, in male infertility [200,201]. CoQ10 has also been shown to reduce FSH and LH levels [202]. It has been discovered that there is a negative correlation between the serum levels of testosterone, E2, fT4, and sperm DNA fragmentation [203,204]. Besides selenium and coenzyme Q10 (CoQ10), N-acetyl-cysteine, in particular, has also been shown to impact semen parameters by increasing the levels of testosterone and inhibin B [205]. Nonetheless, additional research is required to determine relevant antioxidants, as well as their appropriate levels, that could potentially be used in clinical future practice in the treatment of endocrinopathies-induced male infertility [206].

It has been discovered that the administration of FSH can minimize ROS and subsequent sperm DNA damage in idiopathic infertile males [207,208]. Although it has been claimed that testosterone can cause DNA damage in Sertoli and germ cells by activating caspase activity in Sertoli cells, further research is needed to determine whether this is accurate [209]. It has been suggested that the long-term effects of antioxidants can change the levels of FSH, testosterone, and inhibin B [210].

## 12. Conclusions

Endocrine regulations of male reproductive functions follow intricate mechanisms. The principal endocrine axis in the regulation of male reproduction, HPG axis, is subjected to positive and negative feedback regulations by testicular hormones as well as to modulation via various other endocrine axes and numerous other reproductive and nonreproductive hormones. Quantitative or qualitative defects of any of this hormonal crosstalk or their receptors would lead to a spectrum of endocrinopathies, such as hypergonadotropic and hypogonadotropic hypogonadisms, androgen/estrogen excess, and hyperprolactinemia, causing male subfertility or infertility. The precise understanding of the endocrine disruptions-mediated male infertility may pave ways to research unleashing the potential diagnostic tools, offering effective management and treatment protocol to address this multifarious and sensitive pathological state.

## Figures and Tables

**Table 1:** Reports on endocrinopathies and their impact on male reproduction.

Endocrinopathy	Changes in Male Reproduction	Study
Hypogonadotropic hypogonadism (Genetic: Kallman syndrome)	Delayed puberty and infertility caused by a malfunction of GnRH-secreting neurons to migrate; cessation of gonadotropin secretion	[12,14]
Hypergonadotropic hypogonadism	Increased FSH/LH, normal or ↓testis volume, decreased pubic hair and penis size, infertility	[15]
Androgen excess	Inhibition to GnRH secretion, normal or ↓FSH, ↓LH,	[16,17]
Estrogen excess	↓T:E2, ↓semen parameters	[18,19]
Hyperprolactinemia	Normal or ↓FSH/LH, ↓testosterone	[20,21,22,23]
Insulin disorders	↓spermatogenesis, ↓reduced vacuolization in the Sertoli cells, ↓fertility, ↓semen parameters, ↓Leydig cells count, ↓testosterone	[24,25,26,27,28]

↓ = decrease; T:E2, testosterone to estradiol ratio.

**Table 2:** Effects of hypo- and hyperthyroidism on male reproductive functions.

	Hypothyroidism	Hyperthyroidism	References
Prepubertal testicular volume and function	↑ Early onset of spermatogenesis	↓	[75,76,77]
Sperm count	Normal or ↓	↓	[78,79]
Testicular germ cell count	↓		[63,64,71]
Sperm motility	↓	↓	[80,81]
Sexual function	Impaired	Impaired; precocious ejaculation	[73,79,81,82]
Erectile function	↓	↓	[73,82,83]
Free testosterone level	↓	↓	[68]
LH and FSH levels	↓	↑ and SHBG	[69]
E2	↓	↑	[84,85,86]

↑ = increase, ↓ = decrease.

## Data Availability

Not applicable.
